# Transcriptome profile of highly osteoblastic/cementoblastic periodontal ligament cell clones

**DOI:** 10.1590/1678-7757-2020-0242

**Published:** 2020-10-19

**Authors:** Miki Taketomi SAITO, Luciana Souto MOFATTO, Mayra Laino ALBIERO, Márcio Zafallon CASATI, Enilson Antonio SALLUM, Francisco Humberto NOCITI, Karina Gonzales SILVÉRIO

**Affiliations:** 1 Universidade Federal do Pará Instituto de Ciências da Saude Departmento de Saúde Pública Belém Pará Brasil Universidade Federal do Pará, Instituto de Ciências da Saude, Departmento de Saúde Pública, Belém, Pará, Brasil.; 2 Universidade Estadual de Campinas Instituto de Biologia Departamento de Genética e Evolução, Microbiologia e Imunologia Campinas SP Brasil Universidade Estadual de Campinas, Instituto de Biologia (UNICAMP), Departamento de Genética e Evolução, Microbiologia e Imunologia, Laboratório de Genônica e Expressão, Campinas, SP, Brasil.; 3 Universidade de Sorocaba Departmento de Periodontia Sorocaba SP Brasil Universidade de Sorocaba, (UNISO), Departmento de Periodontia, Sorocaba, SP, Brasil.; 4 Universidade Estadual de Campinas Faculdade de Odontologia de Piracicaba Departmento de Prótese e Periodontia Piracicaba SP Brasil Universidade Estadual de Campinas (UNICAMP), Faculdade de Odontologia de Piracicaba, Departmento de Prótese e Periodontia, Divisão de Periodontia, Piracicaba, SP, Brasil.

**Keywords:** Cell differentiation, Clone cells, Sequence Analysis, RNA, Periodontal ligament, Osteoblasts

## Abstract

Heterogeneous cell populations of osteo/cementoblastic (O/C) or fibroblastic phenotypes constitute the periodontal dental ligament (PDL). A better understanding of these PDL cell subpopulations is essential to propose regenerative approaches based on a sound biological rationale.

**Objective:**

Our study aimed to clarify the differential transcriptome profile of PDL cells poised to differentiate into the O/C cell lineage.

**Methodology:**

To characterize periodontal-derived cells with distinct differentiation capacities, single-cell-derived clones were isolated from adult human PDL progenitor cells and their potential to differentiate into osteo/cementoblastic (O/C) phenotype (C-O clones) or fibroblastic phenotype (C-F clones) was assessed *in vitro*. The transcriptome profile of the clonal cell lines in standard medium cultivation was evaluated using next-generation sequencing technology (RNA-seq). Over 230 differentially expressed genes (DEG) were identified, in which C-O clones showed a higher number of upregulated genes (193) and 42 downregulated genes.

**Results:**

The upregulated genes were associated with the Cadherin and Wnt signaling pathways as well as annotated biological processes, including “anatomical structure development” and “cell adhesion.” Both transcriptome and RT-qPCR showed up-regulation of *WNT2*, *WNT16*, and *WIF1* in C-O clones.

**Conclusions:**

This comprehensive transcriptomic assessment of human PDL progenitor cells revealed that expression of transcripts related to the biological process “anatomical structure development,” Cadherin signaling, and Wnt signaling can identify PDL cells with a higher potential to commit to the O/C phenotype. A better understanding of these pathways and their function in O/C differentiation will help to improve protocols for periodontal regenerative therapies.

## Introduction

Periodontitis is a polymicrobial, infection-induced inflammatory disease in the periodontium characterized by connective attachment loss and alveolar bone destruction. Epidemiological studies indicate that periodontitis is still a globally prevalent disease. This periodontal disease may lead to functionally compromised dentition, which affects the quality of life of many subjects.^1^ In the last decade, several attempts have been made to regenerate the tissues impaired due to periodontitis, including bone replacement grafts, guided tissue regeneration, enamel matrix derivative, and combined therapy.^2^ However, these clinical approaches have not shown complete and predictable regeneration of periodontal tissues, namely cementum, periodontal ligament (PDL), and alveolar bone.^2,3^ Therefore, emerging regenerative approaches based on a biological rationale have been proposed to achieve improved clinical outcomes, such as enamel matrix derivative (EMD), recombinant human platelet-derived growth factor-BB (rhPDGF-BB)/beta tricalcium phosphate (b-TCP), and synthetic peptide-binding protein P-15/anorganic bovine bone matrix.^2,3^

Regenerative stem cell therapy has recently gained attention, since postnatal mesenchymal stem/progenitor cells can be isolated from the periodontal ligament and other dental tissues.^2-4^ These progenitor cells have been characterized by the expression of mesenchymal surface markers (CD105, CD146, CD166, CD73, and STRO1), low expression of hematopoietic stem cell markers (CD34 and CD45), and by having stem cell-like properties, including the capacity for self-renewal and multipotency.^2-6^

The periodontium is a complex structure composed of mineralized (cementum and alveolar bone) and non-mineralized tissues (PDL). Consequently, the regeneration of the periodontium requires a well-coordinated process of cell differentiation. However, a detailed understanding of periodontal-derived cells, which is crucial for these emerging approaches, remains unclear.^2,6-7^ It is known that PDL is constituted by heterogeneous cell populations.^2,6,8^ However, the molecular profile that distinguishes cells committed to osteo/cementoblastic (O/C) or fibroblastic phenotypes in PDL is still not fully understood.^2,6,9^ To date, some studies have suggested that cathepsin K is involved in PDL tissue homeostasis through stimulation of collagen fiber accumulation and inhibition of osteoblast differentiation of human PDL cells.^7^ Additionally, evidence suggests that the activation of the canonical Wnt signaling pathway enhances *in vitro* cementoblast differentiation of human PDL cells.^9^

Emerging methods using high-throughput sequencing technologies (such as the massive parallelization of RNA-seq) have broadened our view of the extent and complexity of the PDL transcriptome.^10^ For instance, RNA-seq analysis allows the detection and quantification of a broad range of transcripts and their splice-forms without requiring target specification, which leads to an unbiased and systematic approach to produce insights into important biological pathways and molecular mechanisms for cell regulation in a hypothesis-neutral environment.^10-12^ In our study, CD105-enriched PDL cell clones with osteoblastic/cementoblastic or fibroblastic potential were purified and had their transcriptomes compared after high-throughput RNA sequencing. Our hypothesis is that a comprehensive analysis of periodontium cells may shed light on how to promote an optimal microenvironment for periodontal mineralized and non-mineralized tissue formation. Finally, we expect that our results help in the development of more predictable outcomes for future regenerative approaches.

## Methodology

### Cell culture and flow cytometric analysis

The study design and procedures were approved by the Institutional Review Board of Piracicaba Dental School – State University of Campinas (#053/2013). Unsorted PDL cells and PDL-CD105^+^ enriched populations from permanent teeth were isolated and characterized in a previously published study,^13^ in which participants signed an informed consent form. In short, CD105^+^-enriched PDL cell subsets were obtained using the magnetic cell sorting system (MACS, Milteny Biotech, Germany) following the manufacturer’s recommendations. To confirm the expression of mesenchymal cell-surface markers, flow cytometry was performed as previously described.^13^ The cell suspension was obtained by detaching monolayers of PDL-CD105^+^ cells with 5 mg/mL of Collagenase IV (Gibco, USA) and 5mM EDTA (Applied Biosystems, USA), and resuspended in blocking buffer for 20 minutes with 10% normal donkey serum (Sigma). Cells (1 × 10^6^) were incubated with mouse anti-human monoclonal antibodies against CD105-allophycocyanin (eBioscience, USA), CD146-allophycocyanin (BioLegend, USA), CD166-phycoerythrin (BD Bioscience, USA), CD34-fluorescein isothiocyanate (BD Bioscience, USA), CD45-peridinin chlorophyll (BD Bioscience, USA), Stro-1 Alexa Fluor 647 (BioLegend, USA), or isotype-matched control IgGs /IgM for 40 min at 4°C. A FACScan instrument (BD FACSCalibur™; BD Bioscience Pharmigen, USA) was used for quantitative fluorescence-activated cell sorter (FACS) analysis, and the results were processed using CELLQUEST software (BD Bioscience Pharmigen, USA)

### Cell cloning

As previously described^8^, only one cryovial from the PDL-CD105^+^ cell population was used for cloning isolation through the ring-cloning technique. In total, 250 cells (passage 2) were seeded into 100-mm dishes and incubated at 37°C, 5% CO_2_, in a standard medium composed by Dulbecco’s modified Eagle medium-high glucose (DMEM) supplemented with 10% FBS, penicillin (100 U/ml), and streptomycin (100 mg/mL) (Gibco, USA). Individual clones were allowed to develop for 14 to 21 days until they reached approximately 50 cells per colony. Then, the ring-cloning technique was performed by placing 8-mm-diameter cylinder polystyrene rings (Millipore, USA) around each colony. Lastly, the cells were detached with 0.05% (w/v) trypsin and 0.05 mM (w/v) EDTA (Gibco, USA), transferred to 24-well plates, and recultured as above.

### *In vitro* biomineralization assay

To assess the ability of *in vitro* mineralized matrix formation*,* unsorted PDL cell populations, PDL-CD105^+^ enriched populations, and PDL-CD105^+^ cell clones were seeded (2 × 10^5^cells/well) in 24-well plates for 24 h with standard medium (Control) composed of Dulbecco’s modified Eagle medium-high glucose (DMEM). The medium was supplemented with 10% FBS, penicillin (100 U/ml) and streptomycin (100 mg/mL) (Gibco, USA), and then cells were incubated for 24 h at 37°C and 5% CO_2_. Subsequently, cells were cultivated in fresh standard medium or osteogenic medium (OM), composed of standard medium supplemented with 50 mg/mL ascorbic acid, 10 mM b-glycerophosphate, and 10^-8^ M dexamethasone (Sigma-Aldrich, USA). After 28 days of the induction period, we performed the Alizarin Red staining (AR-S, Sigma-Aldrich, USA) assay as described elsewhere.^14^ Cell clones that formed a mineralized matrix *in vitro* were classified as clones of osteo/cementoblastic (O/C) phenotype (C-O). In contrast, cell clones that could not form a mineralized matrix *in vitro* were named fibroblastic phenotype (C-F).

### Cell Metabolic Activity Assay

For the metabolic analysis, cell clones were seeded (5 × 10^3^ cells/well) in a 96-well plate (Corning Costar, USA) using standard medium and incubated in a humidified incubator at 37°C and 5% CO_2_ for 24 h to allow cell adhesion to the discs. Thenr, the medium was changed for DMEM supplemented with 2% FBS, penicillin (100 U/ml), and streptomycin (100 mg/mL). This time point was considered as the baseline (time 0h) for the metabolic assay. The media was then replaced on days 3 and 7, and the metabolic activity of the cell on the experimental groups was evaluated at days 1, 3, 7, and 10, as previously described^15^ using the 3-(4,5-dimethylthiazol-2-yl)-2,5-diphenyl tetrazolium (MTT, Life Technologies, USA) assay.

### RNA isolation, RNA-seq, and quantitative reverse transcription-polymerase chain reaction (RT-qPCR)

Each cell clone was seeded, and RNA extraction was performed as previously described.^8^ RNA isolated from two C-O clones and two C-F clones cultivated in the standard medium during 14 days were subjected to RNA-seq, and each clone was considered a biological replicate for C-O and C-F group. RNA-seq was performed using Illumina TruSeq RNA Sample Preparation kit v2 (Illumina, USA), according to the manufacturer’s instruction. For RT-qPCR, single-stranded complementary DNA (cDNA) was synthesized from 1 µg total DNA-free RNA using Transcriptor First Strand cDNA synthesis kit (Roche Applied Science, USA) following the manufacturer’s recommendations. RT-qPCR was performed using the samples of cDNA and LightCycler 480 SYBR Green I master kit on the LightCycler 480 II real-time PCR system (Roche Applied Science, USA) for primers sequences *WNT2, WNT2B, WNT16, WIF1, PCDHGA10, BMP4* and *GAPDH* (Table 1). Distilled water (no template control) was used as a negative control for all experiments. Relative quantification of reaction products was accomplished to *GAPDH* and estimated by the ΔCT-method.

**Table 1:** Specific primer sequence for quantitative Polymarase Chain Reaction (qPCR)

Gene ID	Primers (5`® 3`)
GAPDH	Forward: ACATCATCCCTGCCTCTAC; Reverse: CCACCTTCTTGATGTCATCATATTTG
WNT2	Forward: TTTGGCAGGGTCCTACTCC; Reverse: CCTGGTGATGGCAAATACAA
WNT2B	Forward: AACTTACATAATAACCGCTGTGGTC; Reverse: ACTCACGCCATGGCACTT
WNT16	Forward: CAATGAACCTACATAACAATGAAGC; Reverse: CAGCGGCAGTCTACTGACAT
WIF1	Forward: CCAGGGAGACCTCTGTTCAA; Reverse: TTGGGTTCATGGCAGGTT
BMP4	Forward: CTGCAACCGTTCAGAGGTC; Reverse: TGCTCGGGATGGCACTAC
PCDHGA10	Forward: ATTTGCCTGTGGGCACTC; Reverse: CACTTCTCCATTGGCACCTT

### Data analysis

All RNA-seq data generated in our study are available at the GEO repository (http://www.ncbi.nlm.nih.gov/geo/; accession #GSE94599). For RNA-seq data, the quality of raw data was evaluated by *FastQC* (http://www.bioinformatics.babraham.ac.uk/projects/fastqc/). Data were filtered by quality using *Perl* scripts with a 20 quality score threshold. The adapters were removed with Cutadapt^16^ and trim galore (http://www.bioinformatics.babraham.ac.uk/projects/trim_galore/). Filtered reads of the RNA library were mapped against the human genome (GRCh38) using the pipeline *Tophat2-Cufflinks*.^17^ The number of reads aligned per genes and fragments per kilobase of transcript per million mapped reads (FPKM) were estimated by *RSEM* program.^18^ Differentially expressed genes (DEG) between C-O and C-F clones were obtained by *DESeq*^19^ and *EdgeR* packages^20^ (R/Bioconductor) with α=5% and |log2FC|≥1. Heatmaps were then generated based on z-score values estimated from FPKMs values of DEG using the *heatmap* package on R. DEG between C-O and C-F clones were subjected to functional annotation using the DAVID program (Database for Annotation, Visualization, and Integrated Discovery), version 6.8 (https://david.ncifcrf.gov/),^21^ to identify enriched terms of Gene Ontology (GO). GO analysis was generated with the DAVID software based on biological processes (GO_TERM_BP_2 database). Pathway overrepresentation analysis was performed in the Panther Classification System (http://pantherdb.org/). We only considered the enriched GO terms and pathways generated by a modified Fisher Exact test followed by the Bonferroni test and *p*-value threshold of <0.05.

For other experiments, data were expressed as mean ± standard deviation (SD). T-test was used to analyze differences between two groups, and one-way or two-way analysis of variance (ANOVA) (α=0.05) was used to analyze differences among three or factorial analyses, respectively.

## Results

**Figure 1:**
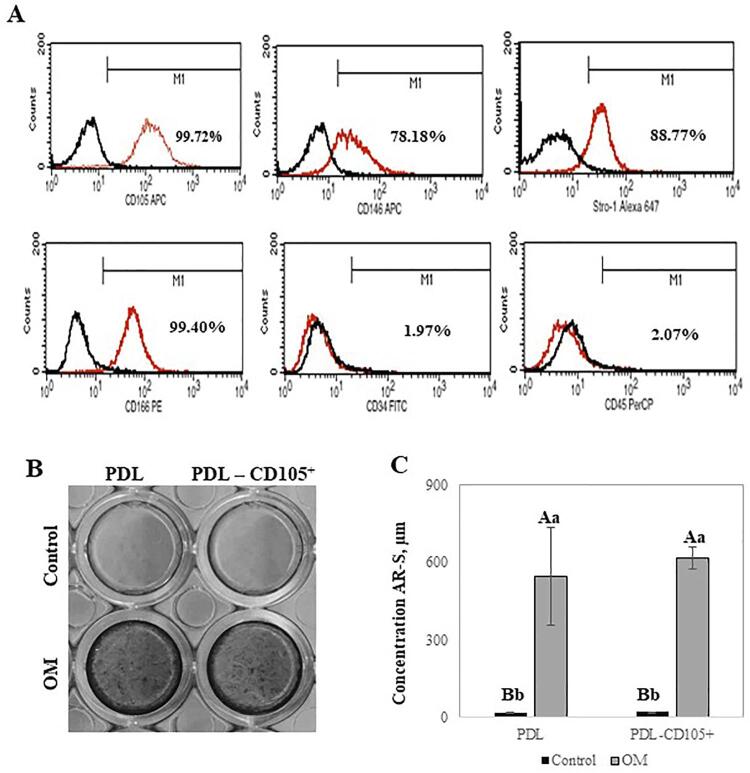
Periodontal ligament derived (PDL) CD105+-enriched population characterization. (A) Flow cytometric analysis of PDL-CD105+-enriched population. The relative levels of cell surface expression were analyzed using CD105, CD146, CD166, STRO-1, CD34 and CD45 antibodies (red histograms) and their isotype-matched antibodies as control (black histograms). (B) Alizarin Red Stain (AR-S) assay demonstrated that PDL-CD105+-enriched population showed similar potential of biomineralization as the unsorted population (PDL) when cultivated under osteogenic induction (OM). (C) Quantification AR-S showed that both PDL-CD105+-enriched population and unsorted PDL presented significant increase of AR-S when cultivated in OM comparing control medium. Bars in (C) represent mean ± standard deviation (SD), in which intragroup statistical differences analysis are indicated by distinct capital letters, and intergroup statistical differences analysis are indicated by distinct lowercase letters. Statistical significance was determined using Two-way ANOVA/Tukey’s test (p<0.05)

Since we aimed to isolate clones with distinct O/C differentiation potentials from a PDL-CD105^+^-enriched population, the biomineralization potential of this population was evaluated and compared to the total pool of cells obtained from PDL from third molars. The PDL-CD105^+^-enriched population exhibited a high proportion of cells that expressed mesenchymal stem cell (MSC)-related markers (Figure 1A). However, the capacity for biomineralization of the PDL-CD105^+^-enriched population was not statistically higher than the unsorted population (PDL) (Figures 1B and 1C).

### PDL clones present distinct ability to form a mineralized matrix *in vitro*

**Figure 2:**
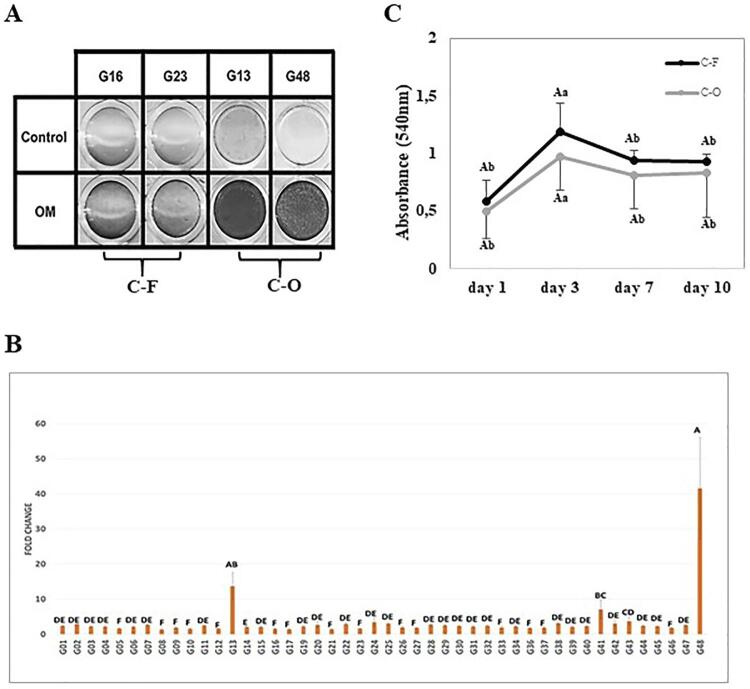
PDL-CD105+- derived cell clones. (A) Alizarin Red staining showed that G16 and G23 clones (C-F cells) could not form mineralized deposits *in vitro*, whereas G13 and G48 clones (C-O cells) showed capacity of biomineralization *in vitro* when cultivated under osteogenic induction (OM). (B) Clones G13 and G48 showed significant quantification of AR-S compared to the other clones. Data are presented as fold increase relative to non-induced controls. Statistical significance was determined using ANOVA/Tukey’s test (p<0.05). (C) Metabolic activity as an indicator for cell viability was measured with MTT assay at the following time points: 1, 3, 7, and 10 days. Distinct capital letters represent intragroup statistical differences, and distinct lowercase letters represent intergroup statistical differences. Statistical significance was determined using Two-way ANOVA/Tukey’s test (p<0.05)

We obtained a total of 46 cell clones from the PDL-CD105^+^ mesenchymal progenitor population. According to the AR-S assay, two of the 46 clones showed a significantly higher potential to form a mineralized matrix *in vitro* under OM induction, namely G13 and G48, and were defined as clones of O/C phenotype (C-O) (Figures 2A and 2B). The remaining 44 clones showed a lower ability to form a mineralized matrix *in vitro* and were classified as clones of the fibroblastic phenotype (C-F). In the C-F group, two clones that rapidly expanded during clonal expansion were selected to represent the C-F group, namely clones G16 and G23 (Figure 2A). The C-F and C-O groups were evaluated for their metabolic activity and showed no significant difference at any time point (Figure 2C). Both clone groups showed increased metabolic activity on day 3 (Figure 2C).

### C-O and C-F clones present different transcriptome profile

**Figure 3:**
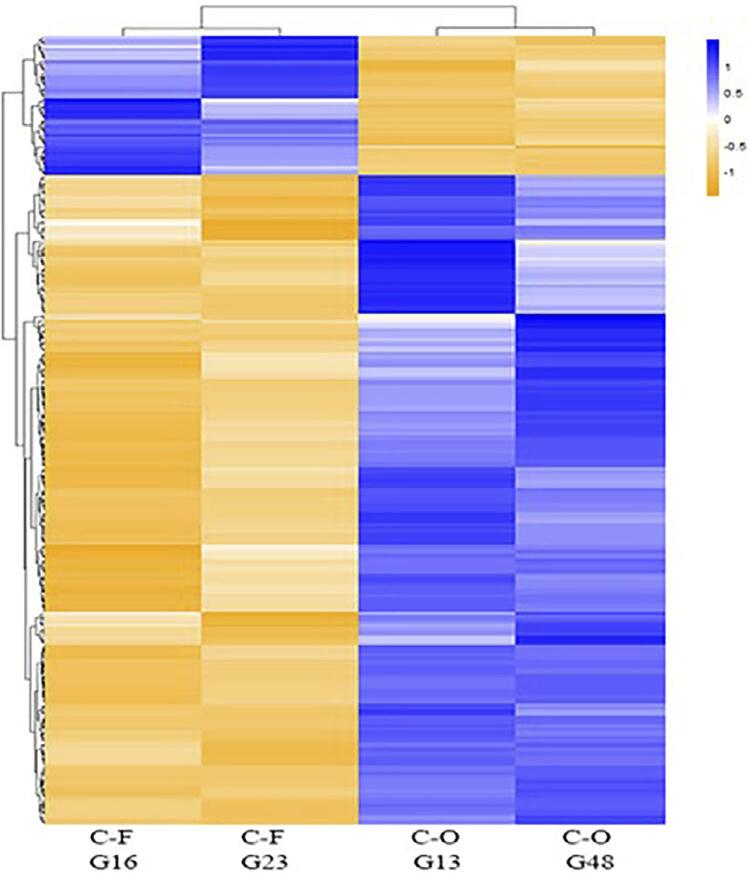
Heatmap of differentially expressed genes of C-O and C-F clones. Differentially expressed genes (DEG) between C-O and C-F clones were obtained by DESeq and EdgeR packages (R/Bioconductor) with α=5% and |log2FC|≥1. Heatmaps were then generated based on z-score values calculated from FPKMs values of DEG using the heatmap package on R. A total of 235 DEG were observed between C-O and C-F clones. In total, 193 genes were significantly up-regulated in C-O group, and 42 genes were significantly down-regulated in C-O group (up-regulated in C-F) (|log2(fold change)|≥1; p<0.05)

All RNA-seq data showed in our study are available at GEO repository (http://www.ncbi.nlm.nih.gov/geo/; accession #GSE94599). *FastQC* analysis showed that ~84% of the reads were at optimum quality and aligned to the human genome. Only aligned reads were retained for further analyses. As the heatmap shows, C-O clones presented a higher transcriptional activity (Figure 3). Out of 235 DEG, 193 were significantly upregulated in the C-O group compared to C-F, and 42 genes were significantly downregulated in the C-O group (Figure 3).

### Functional classification shows distinct profiles of DEG in C-O and C-F clones

To investigate the differences between C-O and C-F clones, DEG were analyzed for pathway overrepresentation. C-O clones showed upregulated genes related to 55 pathways, from which the Cadherin signaling pathway (P00012) and Wnt signaling pathway (P00057) were significantly enriched according to the Panther Classification System (Table 2). Downregulated genes in C-O clones were associated with 10 pathways, but no significantly overrepresented pathway was observed compared to *Homo sapiens* genome background (data not shown).

**Table 2:** Significantly overrepresented pathways and respective upregulated genes in C-O clones

Pathway (code)	p value	Gene ID
Wnt signaling pathway (P00057)	5.70E-04	ADSSL1; PCDH18; PCDHB2; PCDHGA6; PCDHGA10; PCDHGB2; PCDHGB4; PRKCD; PRKCZ; SFRP2; WNT16; WNT2; WNT2B
Cadherin signaling pathway (P00012)	1.73E-03	PCDH18; PCDHB2; PCDHB4; PCDHGA6; PCDHGA10; PCDHGB2; WNT16; WNT2; WNT2B

**Figure 4:**
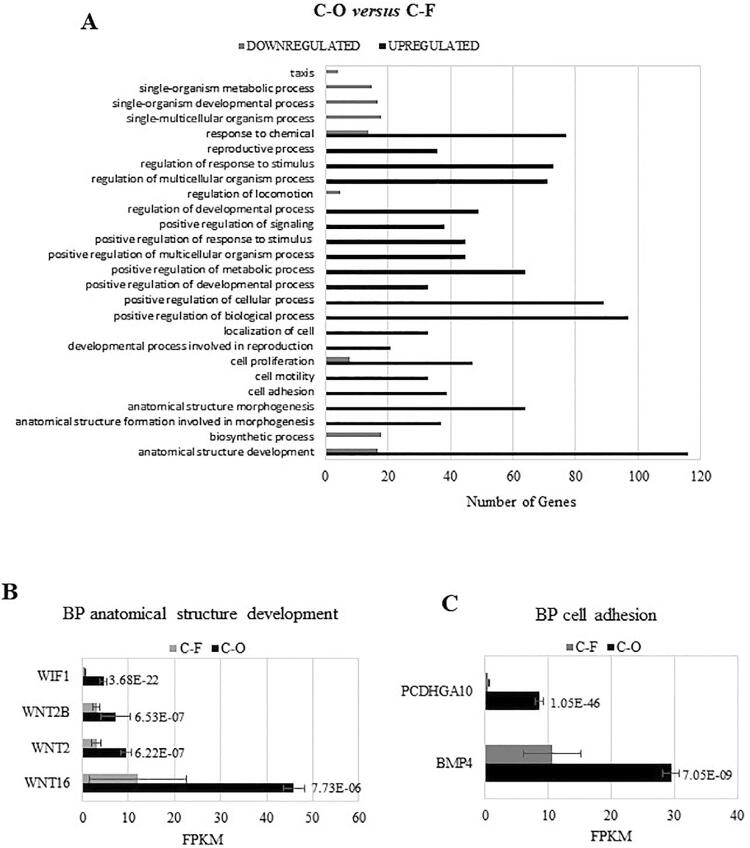
Gene Ontology (GO) of Biological Processes (BP) enrichment analysis of the list of differentially expressed genes between C-O and C-F clones. GO analysis was generated by the DAVID software based on biological processes (GO_TERM_BP_2 database). (A) Number of genes upregulated and downregulated in C-O clones according to GO of Biological Processes. (B) Bar plot of FPKM of upregulated genes in C-O clones for anatomical structure development biological processes. (C) Bar plot of FPKM of upregulated genes in C-O clones for cell adhesion biological processes. Differential expression determined by using of negative binomial generalized linear model (DESeq) in (B) and (C) bar plots

To understand the biological context of DEG, GO analysis was used to map the biological processes enriched in DEG. Genes upregulated in C-O clones showed significant enrichment in 20 biological processes compared to *Homo sapiens* genome background (Figure 4A). Among the significantly enriched biological processes in C-O clones compared to C-F clones, the biological process “anatomical structure development” (GO: 0048856) harbored genes related to the Wnt pathway: *WNT2, WNT2B, WNT16,* and *WIF1* (Figure 4B). Moreover, the biological process “cell adhesion” (GO:0007155) included the gene *PCDHGA10*, which is related to Cadherin pathway, and *BMP4,* which is linked to TGFβ/BMP pathway (Figure 4C).

### *WNT2, WNT16,* and *WIF1 were validated to be upregulated by RT-qPCR*

**Figure 5:**
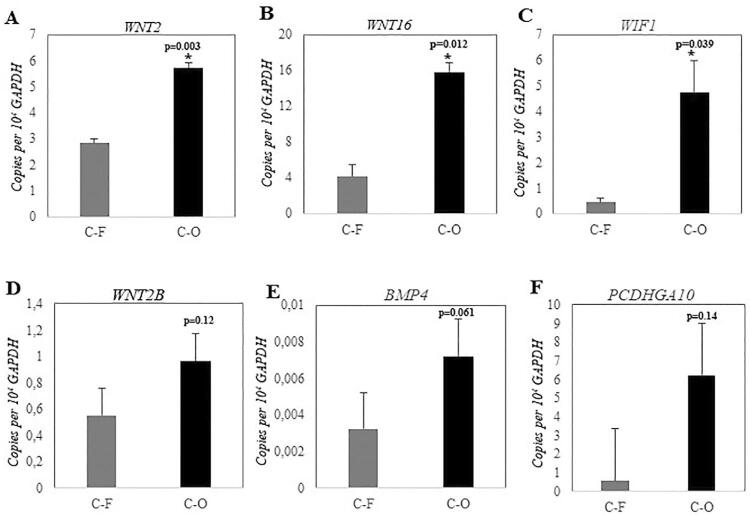
Quantitative reverse transcription-polymerase chain reaction (RT-qPCR) showed that the expression of WNT2 (A), WNT16 (B) and WIF1 (C) was significantly higher in C-O clones than in C-F clones. The expression of WNT2B (D), PCDHGA10 (E) and BMP4 (F) was not statistically significantly up-regulated in C-O clones. Bars represent mean ± standard deviation (SD) of three independent experiments. *Statistical significance intergroup was determined using t-test (p<0.05)

The significantly upregulated genes *WNT2, WNT2B, WNT16, WIF1, PCDHGA10,* and *BMP4,* found after RNA-seq analyses, were then selected for RT-qPCR validation. The data showed that C-O clones presented significantly higher expression of *WNT2, WNT16,* and *WIF1* (Figures 5A-C*).* Although a trend of higher expression in C-O clones was observed, the genes *WNT2B, PCDHGA10,* and *BMP4* presented no significant difference of expression between C-O and C-F clones (Figures 5D-F).

## Discussion

The effective regeneration of the periodontium depends on the formation of mineralized and non-mineralized tissues in an intimate relationship^2^. Although PDL contains heterogeneous cell subpopulations,^6^ the molecular characteristics that distinguish these subpopulations remain unclear.^2^ Attempts to isolate a more purified population by MSCs surface markers, such as CD105 (endoglin), have been reported for periodontal ligament-derived stem cells.^13^ However, the heterogeneity of MSC proliferative and differentiation capacities may not be explained only based on surface markers^8^. Our study showed that PDL-CD105^+^ enriched cell populations and unsorted PDL cells present a similar ability to form mineralized nodules under osteogenic conditions. In agreement with our previous study,^8^ we obtained a PDL-CD105^+^ enriched population that remained with two distinct cell subsets, committed to fibroblastic phenotype (44 of 46 clones) or O/C phenotype (two clones, only). Therefore, since the characteristics of a given cell are determined by patterns of gene expression^7^, we expanded the investigation about PDL-CD105^+^ clones using next-generation sequencing technology to understand what distinguishes a cell line that is more prone to differentiate into an O/C phenotype or to a fibroblastic phenotype.

The use of a label-free approach enables the most inclusive and unbiased description of genes. This approach has been used to describe many types of cells, including osteoblasts^11,12^ and MSC^22^. Despite the existence of previous descriptions of a group of important transcripts to PDL,^7^ high throughput analysis of human PDL clones with distinct differentiation capacity remais unexplored. To the best of our knowledge, this is the first study to analyze primary human PDL cell clones on a comprehensive scale using RNA-seq.

Furthermore, our study showed that, in a heterogeneous cell population derived from PDL, cell clones prone to differentiate into O/C phenotype (C-O clones) have a more active transcriptional profile compared to clones related to fibroblastic phenotype (C-F clones), even when cultivated in standard conditions, such as without osteogenic-inducing mediums. Moreover, genes related to anatomical structure development and Cadherin and Wnt signaling pathways – such as *WNT2, WNT16,* and *WIF1* – allowed us to distinguish PDL cells profile with higher potential to commit to the O/C phenotype.

Wnt signaling is critical for the homeostatic regulation of craniofacial tissue, including PDL and alveolar bone.^23^ Wnt/β-catenin signaling pathway drives mesenchymal progenitor cell differentiation towards osteoblastic phenotype through inhibition of chondroblastic differentiation.^24^ However, after osteoblastic differentiation, the Wnt signaling pathway is downregulated, and the continuous stimulation of this pathway may inhibit mineralization.^25^ In addition, the upregulation of *WNT2* in dental follicles cells at early time points may promote the commitment to O/C progeny before the acquisition of a more mature phenotype.^26^ Furthermore, *WNT16* is associated with regulation of cortical bone homeostasis^27^ and induces expression of osteoprotegerin, which suggests that *WNT16* expression can inhibit osteoclastogenesis.^28^ Accordingly, we observed that periodontal ligament cells more prone to O/C differentiation presented high expression of *WNT2* and *WNT16,* demonstrating the significance of these genes to initial commitment towards the O/C phenotype.

We also found that *WIF1* was one of the most significantly upregulated genes in C-O clones compared to C-F clones. Although Panther analysis did not correlate *WIF1* to the Wnt signaling pathway, previous reports classify this gene as a Wnt antagonist.^29^ In a recent study comparing murine osteoblasts and cementoblasts, the inhibitor of the Wnt pathway, *Wif1,* was found to be upregulated in cementoblasts when compared with osteoblasts and poorly expressed in PDL cells.^30^ Our data suggest that clonal C-O cells are more prone to cementoblastic differentiation; however further investigation is required to confirm this hypothesis.

Wnt pathway alone may not be enough for maturing bone, and other signaling pathways, such as TGF-β/BMPs, and Cadherin pathways may interact with the Wnt pathway to control O/C differentiation.^31^ It has been shown that BMP2 requires the activation of canonical Wnt signaling at the early stage of differentiation of murine dental follicle cell line (SVF4 cells) along O/C phenotype.^32^ Additionally, at the time of follicle cell maturation, BMP2 promotes a negative Wnt-feedback loop by increasing expression of Wnt pathway inhibitors, including *Wif1, Dkk1,* and *Sfrp4*.^32^*BMP4*, another member of TGF-b/BMPs superfamily, also plays an important role in the process of bone nodule formation as already described to *BMP2*.^33,34^ Our results showed that the expression of *BMP4* was constitutively increased about six-fold in C-O compared to C-F cell clones. Similar data were found in immortalized PDL cell clones, in which clones that presented high expression of *BMP4* showed intrinsic ability to form mineralized tissue *in vitro*, whereas another clone that did not express *BMP4* was unable to form mineral nodules.^35^

*BMP4* is considered one of the most predictive gene expression markers of *in vivo* bone formation. Moreover, it has been reported that *BMP4* also interacts with the Wnt signaling pathway during tooth organogenesis.^29,36^ Higher *BMP4* levels are essential to overcome the inhibitory effects of Wnt antagonists, such as *WIF1* and *DKK2*, during tooth development beyond bud stage.^29^ Altogether, we supose that *BMP4* expression in C-O cell clones may be related to the capacity of these clones to acquire the O/C phenotype through functional interactions with the Wnt signaling pathway. However, further experiments are necessary to elucidate how Wnt-BMP interactions affect the O/C maturation process in these cell clones.

The Cadherin superfamily was significantly overrepresented in clones with the potential to differentiate into the O/C phenotype. Studies have reported that the cross-talk between Cadherin and Wnt signaling regulates the mechanism underlining osteoblast differentiation.^37,38^ Cadherins are suggested to bind to β-catenin, hindering its translocation to the nucleus,^38^ thus reducing canonical Wnt signaling. Cadherins also interact with Wnt co-receptor lipoprotein receptor-related protein 5 (*LRP5*),^37^ which is essential to regulate bone mass.^39^ In consistence with these findings, our study showed that C-O clone cells presented nine upregulated genes common between Cadherin and Wnt pathways, suggesting an interaction of these two pathways in the regulation of O/C cell lineage commitment localized into the periodontal dental ligament.

In short, we provided a comprehensive assessment of the transcriptome of human PDL progenitor cell clones with high O/C differentiation potential using a next-generation sequencing technology (RNA-seq). These findings evidence that Wnt pathway-related genes are critical for identifying PDL cells committed towards O/C differentiation. Further studies are necessary to shed light on the mechanisms of action and the extent of this process of differentiation. A better understating of the molecular regulation of PDL cells committed to the O/C phenotype offers the potential to improve protocols for periodontal regenerative therapies.
